# Natural Coumarins as ABC Transporters Modulators: A Key Mechanistic Question for Osteosarcoma Chemotherapy

**DOI:** 10.1002/cbdv.71699

**Published:** 2026-09-20

**Authors:** Shi‐Hong Ren

**Affiliations:** ^1^ Department of Orthopedic Surgery, The First People's Hospital of Wengling (Taizhou University Affiliated Wenling Hospital), School of Medicine Taizhou University, Wenling Taizhou Zhejiang China

Dear Editor,

We read with great interest the recent article by Azadbeigi et al. [1] entitled “Natural Coumarins Galbanic Acid and Auraptene Improved the Efficacy of Alkeran on Human Osteosarcoma Cells by Targeting ABC Transporters,” published in *Chemistry & Biodiversity*. Osteosarcoma is a highly aggressive bone malignancy with limited therapeutic options, mainly due to the development of multidrug resistance mediated by overexpressed ABC efflux transporters, particularly ABCB1 and ABCG2. In this elegant study, the authors identified two natural sesquiterpene coumarins, galbanic acid (GBA) and auraptene (AUR), as promising chemosensitizers that could enhance the antitumor efficacy of Alkeran in human osteosarcoma MG‑63 cells. We highly appreciate their efforts in exploring safe and effective natural adjuvants to overcome chemotherapy resistance. The authors convincingly showed that GBA and AUR alone exhibited no obvious cytotoxicity but acted synergistically with Alkeran to inhibit cell viability and induce apoptosis. Moreover, molecular docking and dynamics simulations suggested that AUR might interact with the nucleotide‑binding domains (NBDs) of ABC transporters, thus interfering with ATP hydrolysis and drug efflux function. These findings provide important insights into developing natural product‑based strategies to improve osteosarcoma treatment.

Nevertheless, we would like to raise a key scientific question that deserves further in‑depth investigation to fully clarify the underlying mechanism. Although the authors demonstrated that GBA and AUR could modulate ABC transporter activity, the mitoxantrone fluorescent efflux assay adopted in their work carries an inherent methodological limitation: mitoxantrone serves as a universal substrate recognized and extruded by both ABCB1 and ABCG2. Mitoxantrone serves as a universal substrate recognized and extruded by both ABCB1 and ABCG2. The cumulative fluorescence signal measured in this assay reflects the combined efflux activity of the two transporters simultaneously, making it impossible to quantitatively separate the individual functional contribution of ABCB1 from ABCG2. The current mitoxantrone‑based efflux assay does not allow discrimination between the specific contributions of ABCB1 and ABCG2. It remains unclear whether these two coumarins preferentially target ABCB1, ABCG2, or function as dual inhibitors, and which transporter isoform plays a dominant role in mediating the observed chemosensitization effect.

We believe that addressing this question will significantly strengthen the mechanistic conclusions of this valuable study. Using isoform‑specific inhibitors, such as verapamil for ABCB1 and Ko143 for ABCG2, or gene knockdown/knockout models would help define the exact target spectrum of GBA and AUR [2, 3]. Clarifying the transporter specificity will not only deepen our understanding of how these natural coumarins reverse multidrug resistance but also facilitate the rational design of more potent and selective ABC modulators for future preclinical and clinical applications. We sincerely look forward to subsequent studies that will further elucidate this important mechanistic detail and translate these promising findings into clinical benefits for osteosarcoma patients.

## Author Contributions

**Shi‐Hong Ren**: conceptualization, writing – review and editing, writing – original draft, funding acquisition.

## Funding

This work was supported by the General Project of Zhejiang Provincial Health Commission (No. 2024KY548).

## Ethics Statement

This manuscript only conducts academic discussion on published literature, without involving human subjects, animal experiments, or clinical sample collection, so no ethical approval documents are required.

## Conflicts of Interest

The authors declare no conflicts of interest.

## Data Availability

No datasets were generated or analyzed during the current study.
